# Visual assessment versus computer-assisted gray scale analysis in the ultrasound evaluation of neonatal respiratory status

**DOI:** 10.1371/journal.pone.0202397

**Published:** 2018-10-18

**Authors:** Francesco Raimondi, Fiorella Migliaro, Luisa Verdoliva, Diego Gragnaniello, Giovanni Poggi, Roberta Kosova, Carlo Sansone, Gianfranco Vallone, Letizia Capasso

**Affiliations:** 1 Division of Neonatology, Section of Pediatrics, Department of Translational Medical Sciences, Università “Federico II”, Naples, Italy; 2 Department of Electrical Engineering and Information Technology, Università “Federico II”, Naples, Italy; 3 Department of Advanced Biomedical Sciences, Università “Federico II”, Naples, Italy; National Yang-Ming University, TAIWAN

## Abstract

**Background and aim:**

Lung ultrasound has been used to describe common respiratory diseases both by visual and computer-assisted gray scale analysis. In the present paper, we compare both methods in assessing neonatal respiratory status keeping two oxygenation indexes as standards.

**Patients and methods:**

Neonates admitted to the NICU for respiratory distress were enrolled. Two neonatologists not attending the patients performed a lung scan, built a single frame database and rated the images with a standardized score. The same dataset was processed using the gray scale analysis implemented with textural features and machine learning analysis. Both the oxygenation ratio (PaO2/FiO2) and the alveolar arterial oxygen gradient (A-a) were kept as reference standards.

**Results:**

Seventy-five neonates with different respiratory status were enrolled in the study and a dataset of 600 ultrasound frames was built. Visual assessment of respiratory status correlated significantly with PaO2/FiO2 (r = -0.55; p<0.0001) and the A-a (r = 0.59; p<0.0001) with a strong interobserver agreement (K = 0.91). A significant correlation was also found between both oxygenation indexes and the gray scale analysis of lung ultrasound scans using regions of interest corresponding to 50K (r = -0.42; p<0.002 for PaO2/FiO2; r = 0.46 p<0.001 for A-a) and 100K (r = -0.35 p<0.01 for PaO2/FiO2; r = 0.58 p<0.0001 for A-a) pixels regions of interest.

**Conclusions:**

A semi quantitative estimate of the degree of neonatal respiratory distress was demonstrated both by a validated scoring system and by computer assisted analysis of the ultrasound scan. This data may help to implement point of care ultrasound diagnostics in the NICU.

## Introduction

Lung ultrasound (LUS) is attracting a growing interest to describe common respiratory diseases [1]. Unlike other organs, LUS relies on both real anatomic structures (e.g. the pleura) and artifacts (i.e. visual features that do not correspond to true body formations). In the adult patient with interstitial syndrome, vertical hyperechoic artifacts also known as B-lines can be demonstrated. It is currently debated whether the number of B lines is related to the severity of the disease [2]. Neonatologists have used lung ultrasound to characterize meconium aspiration syndrome [3], pneumothorax [4], transient tachypnea of the neonate or respiratory distress syndrome [5]. Besides describing diseases, LUS has a potential in the follow-up of neonatal respiratory distress, though the first attempts in this direction have yielded poor results [6]. Recently, visual scores have been described to correlate ultrasound pictures with the severity of neonatal respiratory distress [7]. While this approach is clinically useful in predicting the need of respiratory support [7–9], it has not yet been tested on neonates on prolonged mechanical ventilation and results may depend on the observer’s expertise. To overcome the latter limitation, quantitation of lung disease severity has been attempted by computer-assisted gray scale analysis in the adult patient [10]. In the present paper, we compare the latter technology, improved through the inclusion of textural features and machine learning analysis, to an ultrasound visual score in evaluating lung scans of preterm infants with variable respiratory status as assessed by blood gases indexes.

## Patients and methods

This prospective, observational investigation was conducted from May 2016 to May 2017 in a level III hospital with 2500 total births per year. The present investigation was approved by the local Institutional Review Board (Comitato Etico "Carlo Romano" presso AOU Federico II), and all clinical investigation have been conducted according to the principles expressed in the Declaration of Helsinki; formal consent was obtained from the parents. We enrolled in the study neonates admitted to the NICU with respiratory distress, defined as tachypnea (i.e. a respiratory rate above 60/minute), chest retractions, nasal flaring and grunting. Major malformations (e.g., congenital diaphragmatic hernia, pulmonary adenomatoid malformation) were considered valid exclusion criteria.

### LUS visual score and analysis

**Fig 1:**
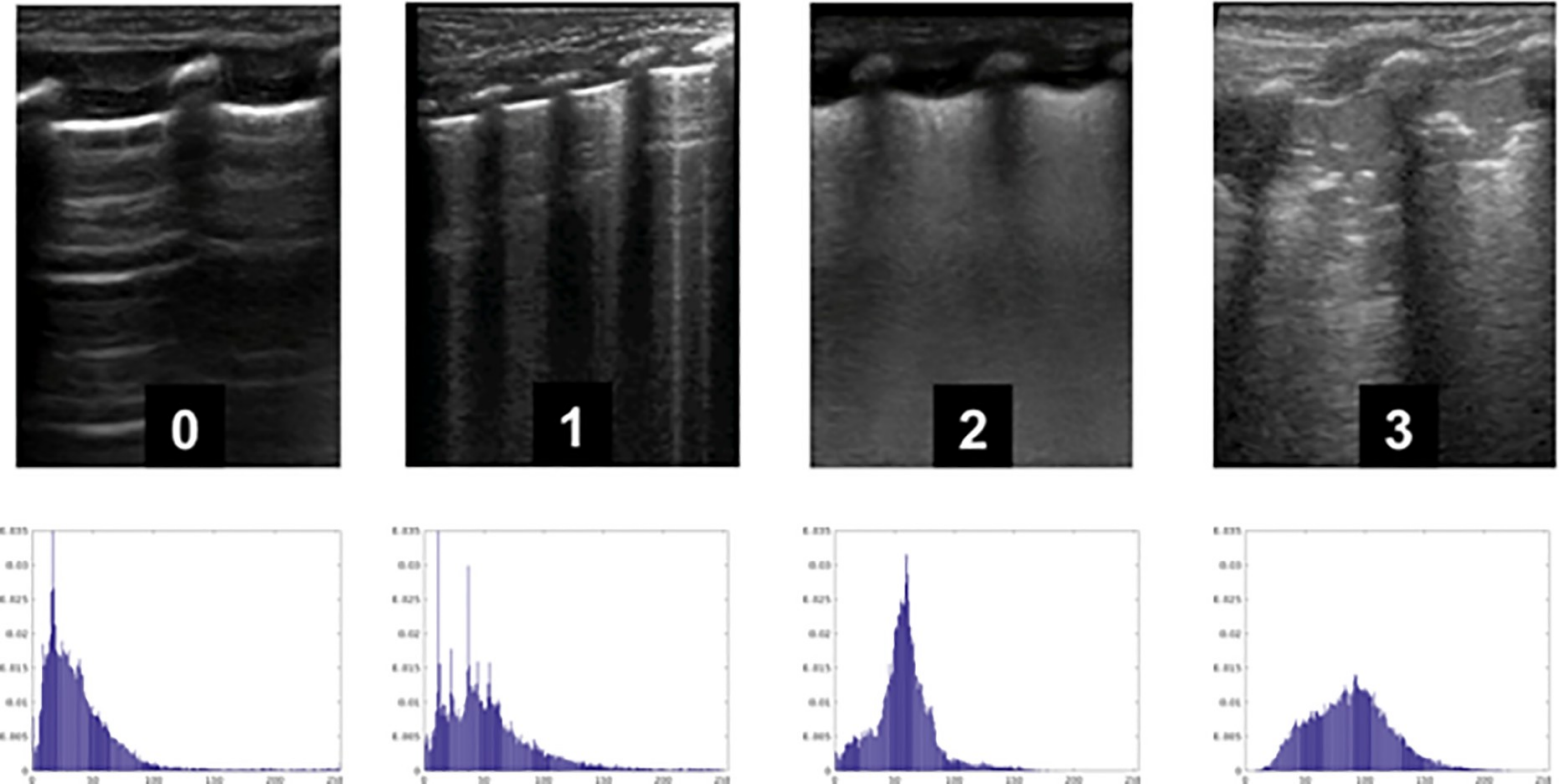
Representative lung ultrasound images used for scoring. 0 Normal pattern with horizontal reverberation of the pleural line (also known as A lines). 1 Vertical hyperechoic artifacts (also known as B lines) more than 3 per field, well spaced. Thin, regular pleural image. 2 Coalescent B lines, thick pleural image with or without small subpleural consolidations. 3 Thick and irregular pleural image with evident subpleural consolidations1.

A broadband linear transducer (mod L12-5, Philips, Eindhoven, the Netherlands) was used to obtain short clips in four standard views (emiclavear, anterior axillary, median axillary, posterior axillary) per side. A single stillframe per clip was extracted as uncompressed DICOM format by a masked operator (D.G.) and eight frames per patient were evaluated by both visual scoring and gray scale analysis. The former was modified from Brat et al [7] attributing a zero to three score to each frame as in Fig 1.

Two neonatologists with different degree of experience (F.M. and R.K) in lung ultrasound and unaware of the patient conditions independently scored the images. A sum value from the 8 scores per patient was used for correlations with oxygenation indexes.

### Computer assisted gray scale analysis

The same still frame dataset was independently assessed by a masked operator (L.V.) by a gray-scale analyzer. To this aim, a dedicated software was developed using the MATLAB® scientific programming language. In particular, an easy to use graphical interface was built, in order to carry out the textural analysis. Specifically, the evaluation of statistics could be performed in different modalities: row-wise, column-wise, frame-wise, but also in running-window modality and finally on a region of interest (ROI) selected by the user using the mouse. Basic functionalities of MATHLAB were integrated in the developed visualization tool. It is worth noting that this tool performed the same statistical image analysis than the software *QUANTA*^*TM*^ *Critical Care*, (CAMELOT Biomedical Systems Srl, Genoa, Italy), allowing to reproduce the same type of experiments carried out by Corradi et al [10,11]. The ROI was selected as to include the pleural line and the area beneath. We considered two approaches: 1) a simpler analysis based on the computation of global and local first-order statistics (i.e. gray-level histogram, mean, variance); and 2) a more advanced analysis accounting for second-order statistics, based on a set of textural features extracted from the Gray-Level Co-occurrence Matrix (GCM) of the data. A first group of 10 such features includes the classical texture descriptors proposed in [12], like contrast, energy, entropy and homogeneity. A second group comprises 7 features that are defined by means of the occurrences of the sum or difference between two gray levels [13,14]. The last group includes 5 correlation-based textural descriptors [12,13]. These features capture the gray-level spatial dependencies among neighboring pixels and are particularly suited to describe the local micro-pattern and macro-pattern variations present in the image (the complete list of all the features can be found in the Appendix).

In order to obtain more powerful textural descriptors, the GCM is usually computed along different directions. In our experiments, we analyzed the co-occurrences of 2-pixel spaced gray-levels along the horizontal and vertical direction, thus obtaining a 44-dimensional feature vector.

**Fig 2:**
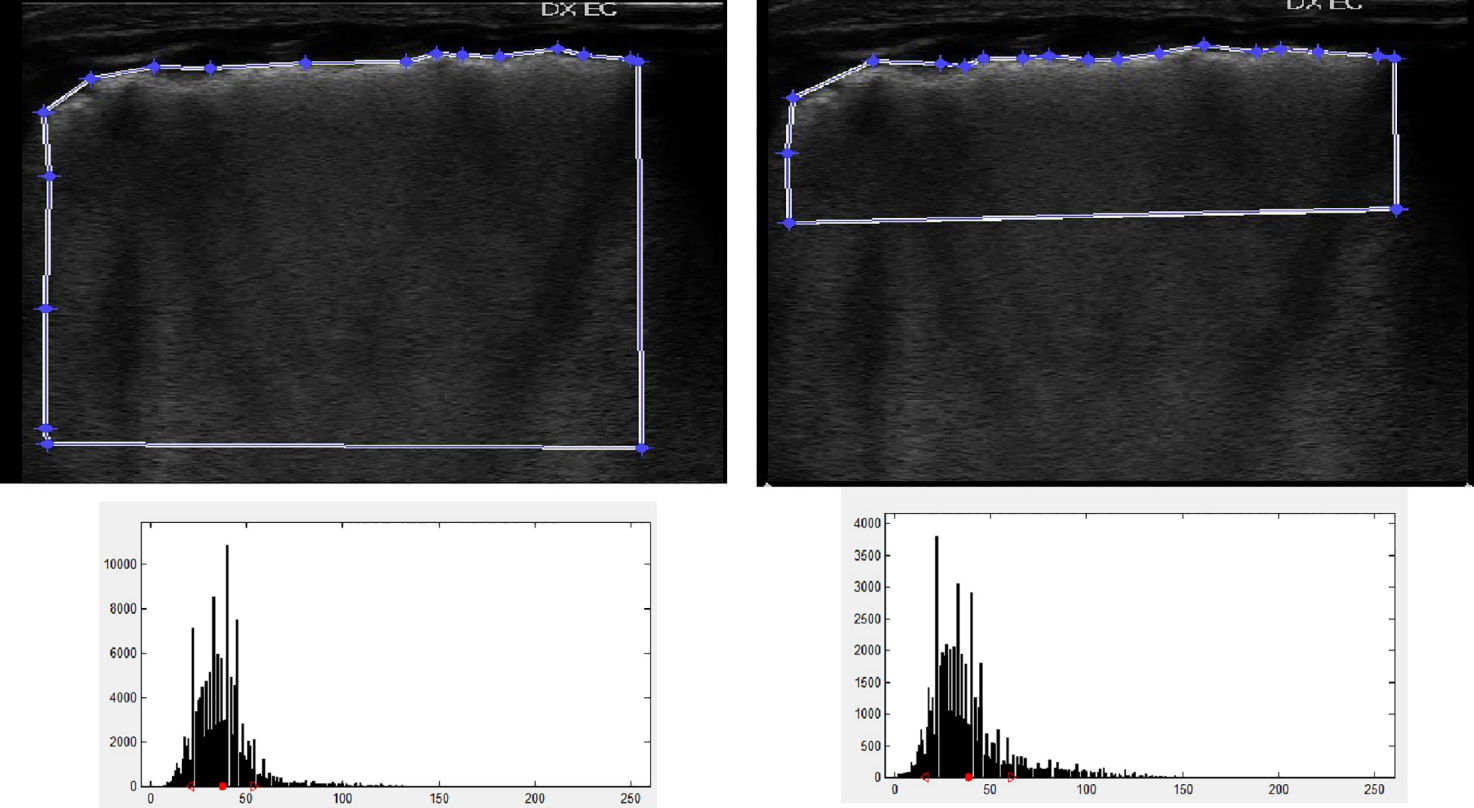
First-order statistics analysis showing ROI distributions (upper panels) and the calculated intensity histograms (lower panels).

The classification step was performed differently for the two approaches. In particular, the eight mean intensity values per patient were pooled into an average value, generating a gray-scale mean intensity score, to be correlated to both oxygenation indexes. Instead, for the textural features we built a Support Vector Machine regressor properly trained on the dataset and carried out a leave-one-patient-out cross validation. The ROI upper limit was drawn by hand following the entire pleural surface. The lateral and bottom sides were then drawn by the computer keeping square angles and constant area of 50 and 100 K pixels, respectively. The rationale was to gain in both cases the maximal amount of information from the subpleural region (where the ultrasound penetration is higher). The 50 and 100 K pixels then differed for the data coming from deeper lung areas. (Fig 2).

Blood gases indexes were:

1. Oxygenation ratio i.e. PaO2 to FiO2;
2. Alveolar-arterial oxygen gradient i.e. A-a gradient = PA − PaO2, where PA indicates alveolar partial pressure and is given by (FiO2 × [760 − 47]) − (PaCO2/0.8);

The purpose of the study was to comparatively correlate the LUS score and the mean grayscale intensity with the oxygenation indexes.

### Statistics

All variables were expressed as mean± standard deviation (SD) or percentage (%). The normality of sample distribution was verified by applying Shapiro-Wilk test. Concordance between operators was analyzed by Cohen test. Correlation between the LUS score or the mean echo intensity (gray units) and the oxygenation indexes was evaluated with Spearman rank test. Statistical significance was assumed with two-tailed *P* values< .05. Statistical analysis was carried out using SPSS version20.0 (SPSS Inc., Chicago, IL).

## Results

A total of 600 frames were recorded from 75 patients with variable respiratory status whose demographics are shown in Table 1.

**Table 1:** Main demographic variables of the study cohort.

	N = 75
Birthweight (grams)	1380 ±681
Gestational age (weeks)	31± 3
Gender	42 F/33M
Antenatal Steroids	41
Patients on mechanical ventilation	49
Patients on invasive support	14
Other or no respiratory support	3
PaO2/FiO2	214 ± 108
Arterial-alveolar gradient (mmHg)	98.5 ± 84.4
RDS	66
TTN	5
EOS	1
Bronchiolitis	2

RDS respiratory distress syndrome; TTN transient tachypnea of the neonate

EOS early onset sepsis

**Fig 3:**
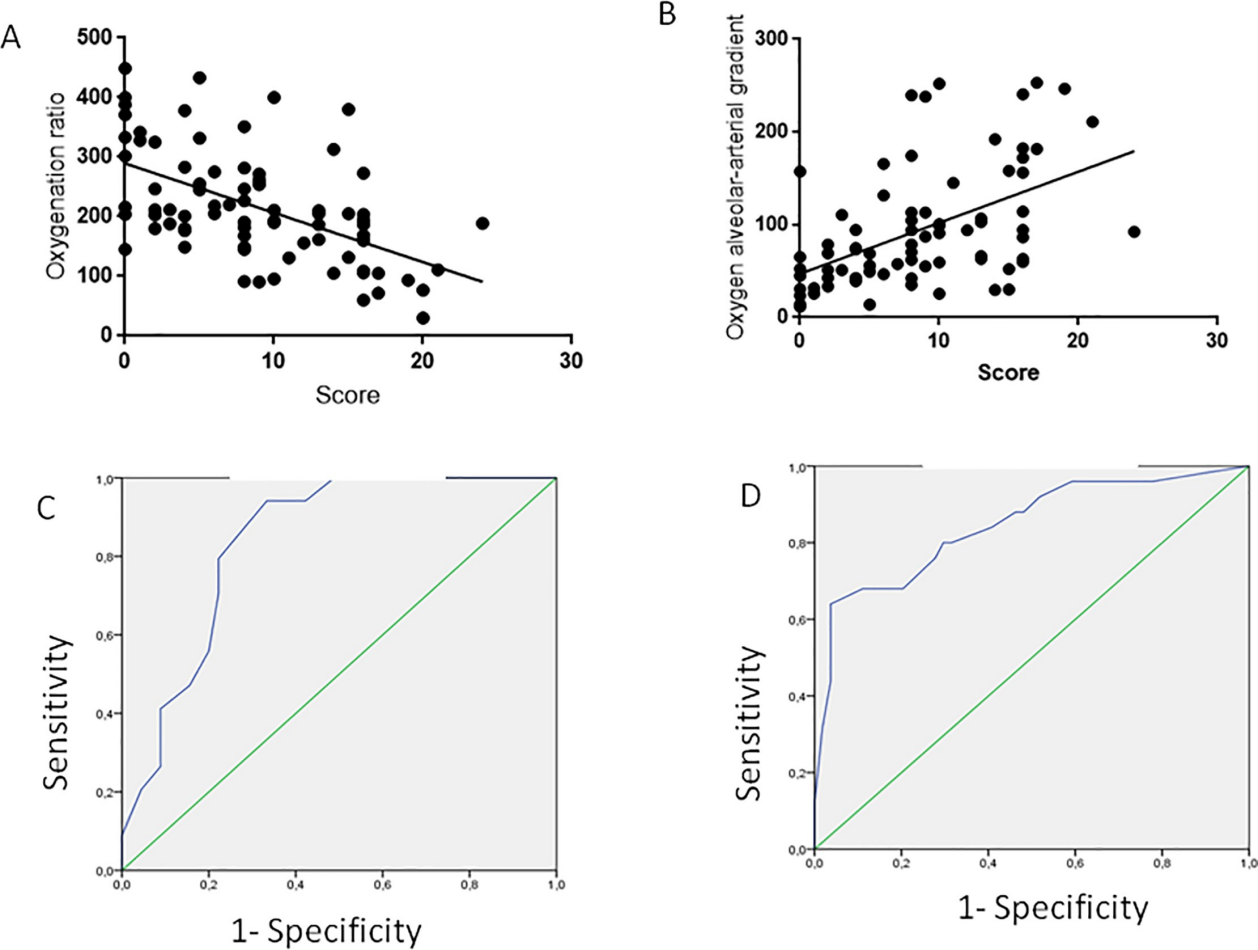
Correlation of visual LUS score with the PaO2/FiO2 ratio (3A); its ROC curve for a cut off value of less than 200 gave an AUC = 0.83 (3C). The correlation of visual LUS score with alveolar arterial gradient is shown in panel 3B; its ROC curve for a cut-off value of more than 150, shown in panel 3D, gave an AUC = 0.844.

The visual LUS score significantly correlated with PaO2/FiO2(r = -0.55; 95% C.I. = -0.68 to -0.35; p<0.0001) and with the A-a gradient (r = 0.59; 95% C.I. = 0.41 to 0.69; p<0.0001) (Fig 3).

**Fig 4:**
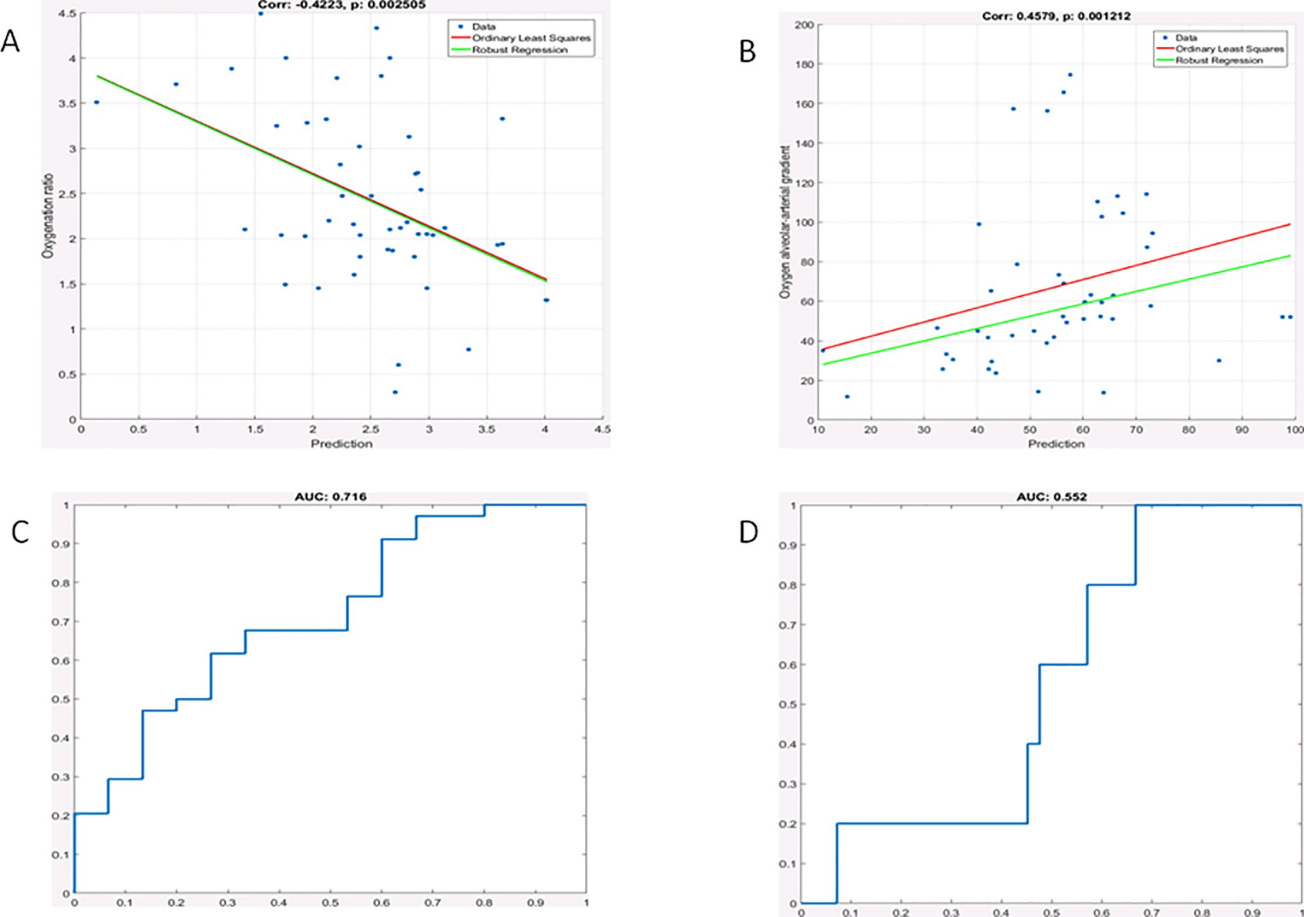
Gray scale analysis results for small region of interest (50K pixels). Correlation of the gray scale analysis with the PaO2/FiO2 ratio (3A); its ROC curve for a cut off value of less than 200 had an AUC = 0.71 (3C). The correlation of the gray scale analysis with the alveolar arterial gradient is shown in panel 3B; its ROC curve for a cut-off value of more than 150, shown in panel 3D, resulted in an AUC = 0.55.

**Fig 5:**
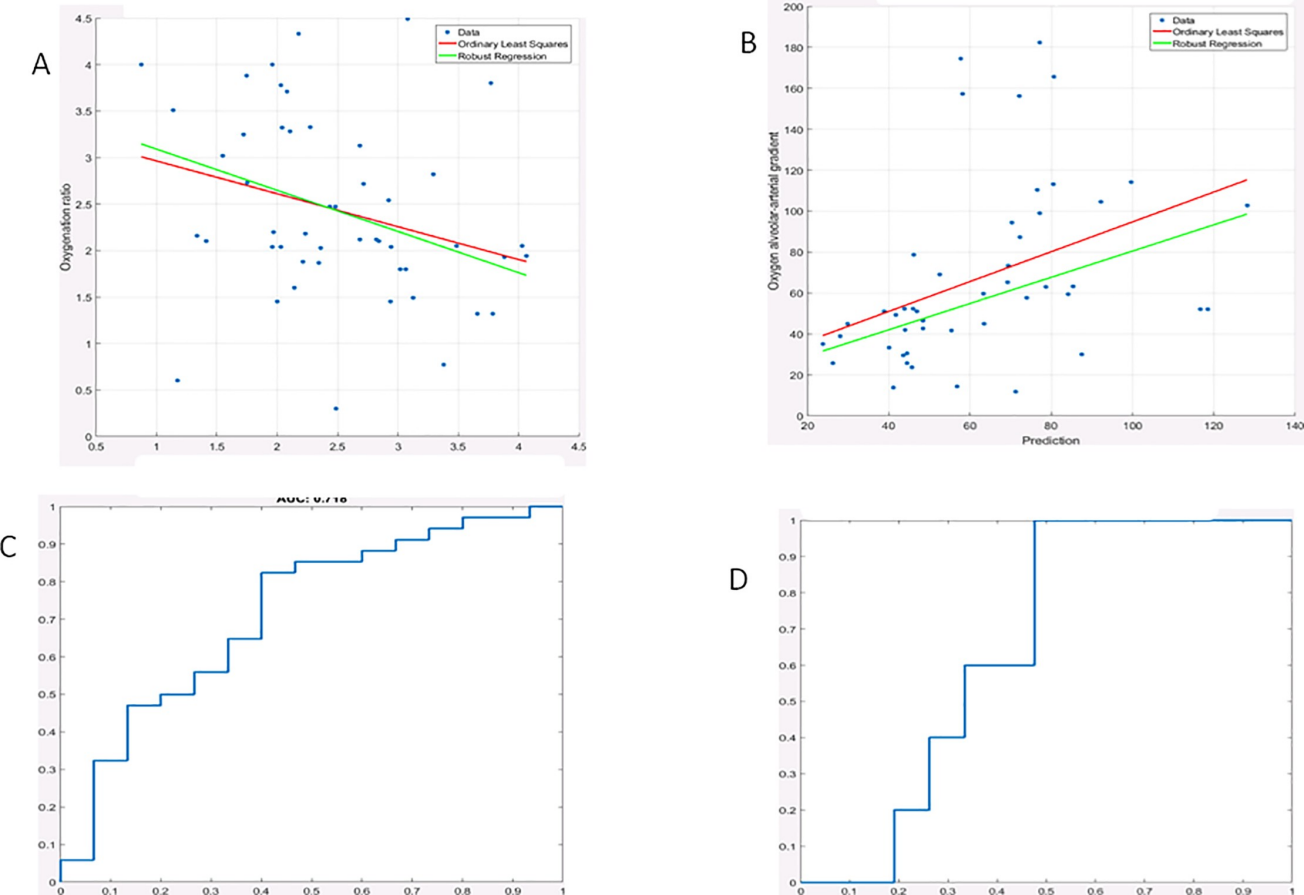
Gray scale analysis results for large region of interest (100K pixels). Correlation of the gray scale analysis score with the PaO2/FiO2 ratio (3A); its ROC curve for a cut off value of less than 200 had an AUC = 0.72 (3C). The correlation of the gray scale analysis with the alveolar arterial gradient is shown in panel 3B; its ROC curve for a cut-off value of more than 150, shown in panel 3D, gave an AUC = 0.66.

The gray scale analysis also correlated with the PaO2/FiO2 ratio and with the A-a gradient (Fig 4) considering a 50k pixel region of interest. When the latter was increased to 100 K pixel, the correlation was significant for both the PaO2/FiO2 ratio and the A-a gradient with comparable strength (Fig 5).

In order to better understand the importance of the textural features, we analyzed the behavior of the three groups of features. In Table 2 we report the results in terms of AUC separately for each group of features and for the whole set of 44 features. Using all features guarantees the best performance in most of the cases, but not always. For example, on the A-a gradient, group 2 provides the best performance with the small ROI, and group 3 with the large ROI. Nonetheless, no single group is uniformly better, and using all features appears to be the most robust choice. As for the impact of the ROI on performance, the correlation with alveolar gradient improves when a larger ROI is adopted, while the correlation with the oxygenation ratio seems to be less sensitive to the ROI size.

**Table 2:** Performance (AUC) for selected groups of features with small and large ROI.

AUC	50k pixel ROI		100k pixel ROI	
Feature group	PaO2/FiO2	A-a gradient	PaO2/FiO2	A-a gradient
1	0.575	0.614	0.724	0.586
2	0.647	0.652	0.710	0.538
3	0.663	0.538	0.626	0.695
All	0.716	0.552	0.718	0.652

**Fig 6:**
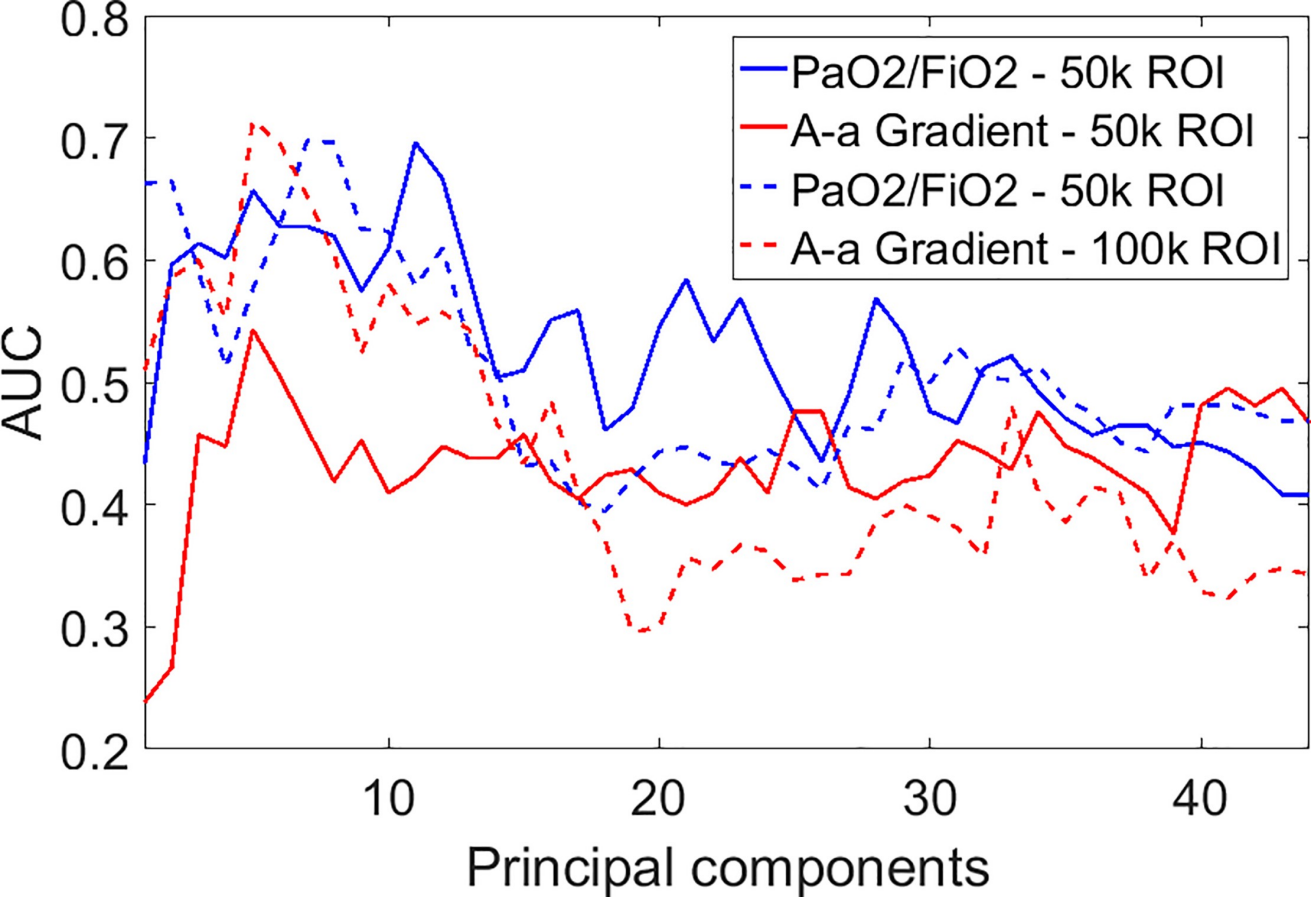
Performance (AUC) for both indexes and ROI sizes as a function of the number of principal components kept in the feature vector. Components are sorted by descending variance.

**Fig 7:**
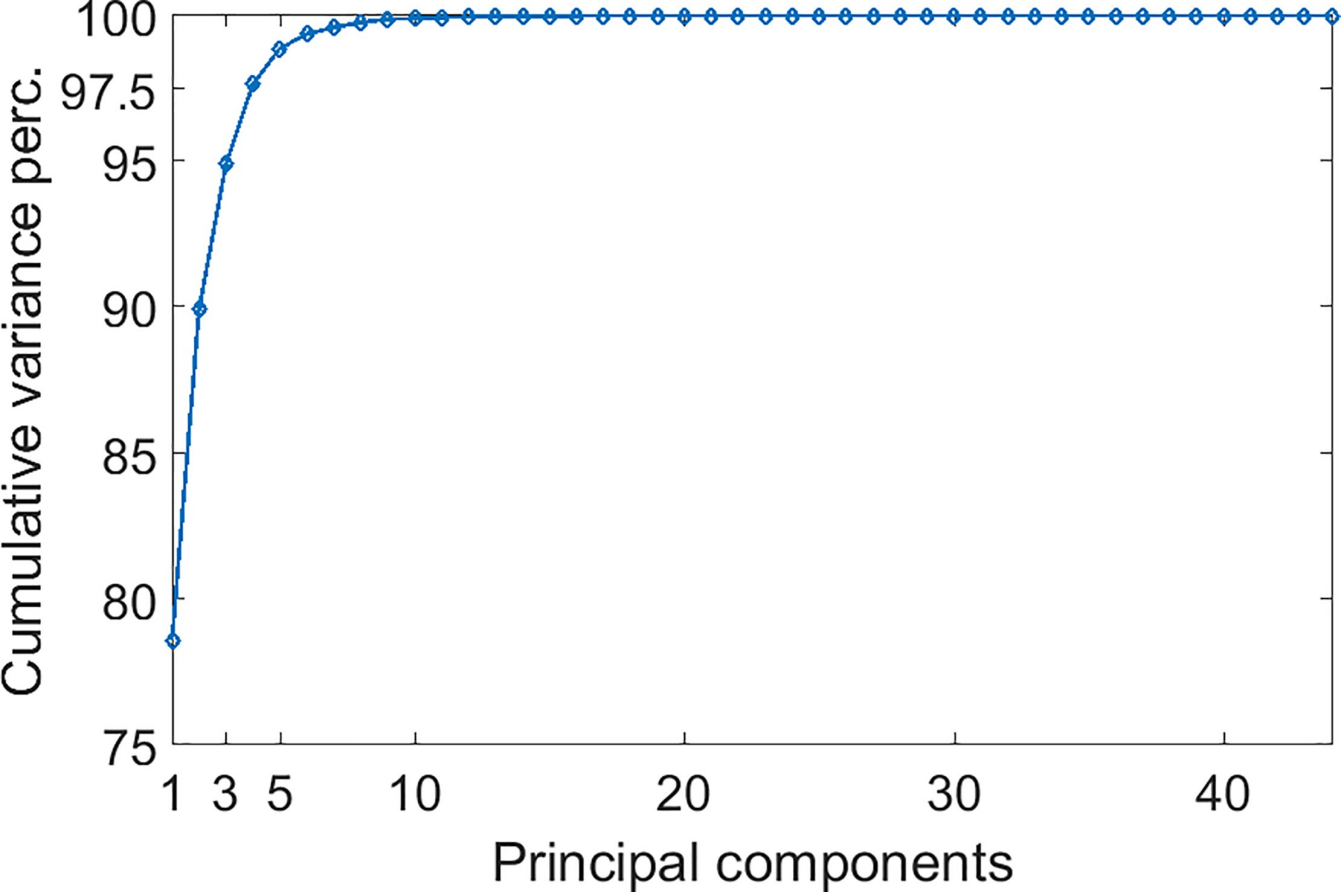
Fraction of the total variance of the full feature vector explained by the first principal components. Components are sorted by descending variance.

To investigate the effects of feature reduction, we carried out the Principal Component Analysis (PCA) of all the features, with the aim to keep only most important in the feature vector. In Fig 6 we show the AUC as a function of the number of principal components (sorted by descending variance) kept in the feature vector. In all cases, the best performance is obtained using only a few principal components, no more than 11. On the other hand, these components account for almost all the variance of the feature vector as shown in Fig 7. For example, the first 3 components explain the 95% of the total variance, and the first 10 reach the 99%

## Discussion

Our results show that a visual assessment and the gray scale analysis of a lung ultrasound database have a significant linear correlation with the oxygenation status in our population of neonates with a variable degree of respiratory distress of diverse origin. An ultrasound score is an appealing tool to monitor the course of significant respiratory distress. Brat et al. had previously described a correlation between lung ultrasound scores and oxygenation status in a cohort of preterm babies mostly on non-invasive respiratory support [7]. They divided each lung in three sections (upper anterior, lower anterior and lateral) using a linear microprobe. We present a modified score using a high frequency, full size, linear transducer that grants at a glance a complete sagittal scan of the neonatal lung. We also extended the investigation to include mostly infants on mechanical ventilation, a population that would greatly benefit from a novel monitoring technique. Both studies agree on the very limited interobserver variability; the different degree of experience of our operators reaffirms the steep learning curve already described by other investigators [15].

The present study is also the first endeavor to quantify neonatal lung ultrasound with a computer assisted technique. In the fetus, a similar strategy has been described by Bonet Carne et with quantitative texture analysis of lung ultrasound images. They conclude that their prenatal estimate of lung maturity by this technique is able to predict neonatal respiratory morbidity with an accuracy comparable to that of validated amniotic fluid tests [16]. In the adult, computer assisted ultrasound quantification has been described for a wide array of pulmonary diagnoses. Raso et al graded pulmonary fibrosis and lung edema by computer analysis in two sets of patients who had been preselected by an expert in lung ultrasound [17]. Corradi et al found that the mean gray scale intensity was more accurate than visual ultrasound assessment in the diagnosis of community acquired pneumonia [10]. The same group later showed that mean intensity correlated with the degree of pulmonary edema in mechanically ventilated cardiac surgery patients [11]. In all adult studies a low frequency sector probe (2.5–3.5 MHz) with a focus in the parenchymal region was used to scan a wide region of interest. In our setting, computer assisted gray scale analysis on first-order statistics *per se* had a poor performance (data not shown) that was significantly improved including textural features and machine learning analysis. Since the width of the region of interest did not significantly modify the results, we speculate that the most superficial sections of the lung- and the pleural line in particular- might be critical for the computer aided analysis. Unlike the artifacts generated in the deep regions of the lung, the pleura is a real anatomic structure. Because of its superficial position in the neonatal chest with thin subcutaneous tissue, the pleura can be studied in good detail with a high frequency transducer. An irregular pleural image is a mandatory ultrasound sign in infants with respiratory distress syndrome [18]. The importance of the pleura was also recently highlighted by Cisneros-Velarde et al assessing the performance of computer-aided diagnosis of pediatric pneumonia [19]. In the future, better results may be achieved with more sophisticated computer assisted study of the pleura. Recently, Veeramani and Muthusamy proposed a classification system of neonatal respiratory disease based on local feature extraction and multi-level relevance vector machine classifier [20].

We acknowledge some limitations to the present pilot study. First, the relatively small number of enrolled newborns with a different origin of respiratory distress and a variable postnatal age led to a wide distribution of the experimental points. A larger dataset with more homogeneous patients may obviate this problem. Second, the study was conducted on a single ultrasound machine by operators working in the same neonatal intensive care unit. Extending the study to a multicenter collaboration may strengthen our results.

In conclusion, our data show that visual assessment and the gray scale analysis correlate with the respiratory status in a population of sick neonates. These novel techniques offer a non-invasive, radiation-free approach to monitoring neonatal lung disease.

## Appendix

Given a gray-scale image quantized with *L* gray levels, the gray-level co-occurrence distribution for a given offset among pixel pairs, is given by:

$$P_{ij}=\frac{N_{ij}}{\sum_{i,j=1}^{L}N_{ij}},$$

where *i* and *j* are two gray-levels, L is the number of gray-levels, and *N*_*ij*_ is the number of pixels displaced by the given offset whose gray-levels are respectively *i* and *j*.

Entropy:

$$H=-\sum_{i,j}P_{ij}\log P_{ij}$$

Inverse difference:

$$INV=\sum_{i,j}\frac{P_{ij}}{1+|i-j|}$$

Homogeneity:

$$HO=\sum_{i,j}\frac{P_{ij}}{1+{\left(i-j\right)}^{2}}$$

Dissimilarity:

$$D=\sum_{i,j}|i-j|P_{ij}$$

Cluster shade:

$$CS=\sum_{i,j}{\left(i+j-\mu_{x}-\mu_{y}\right)}^{3}P_{ij}$$

Cluster prominence:

$$CP=\sum_{i,j}{\left(i+j-\mu_{x}-\mu_{y}\right)}^{4}P_{ij}$$

Correlation:

$$C1=\sum_{i,j}\frac{(i-\mu_{x})(j-\mu_{y})P_{ij}}{\sigma_{x}\sigma_{y}}$$

$$C2=\sum_{i,j}\frac{(ij)P_{ij}-\mu_{x}\mu_{y}}{\sigma_{x}\sigma_{y}}$$

Note that μ and σ are respectively the mean and the standard deviation of the rows (*μ*_*x*_,*σ*_*x*_) and the columns (*μ*_*y*_,*σ*_*y*_) of the marginal distributions of *P*_*ij*_.

Autocorrelation:

$$AC=\sum_{i,j}(ij)P_{ij}$$

Contrast:

$$CO1=\sum_{n=0}^{L-1}n^{2}\sum_{\begin{array}{c} i,j=1\\ |i-j|=n \end{array}}^{L}P_{ij}$$

$$CO2=\sum_{i,j}{\left(i-j\right)}^{2}P_{ij}$$

Energy (or Uniformity or Angular second moment):

$$A=\sum_{i,j}{P_{ij}}^{2}$$

Variance (or Sum of squares):

$$V=\sum_{i,j}{\left(i-\mu \right)}^{2}P_{ij}$$

Sum of average:

$$SA=\sum_{n=2}^{2L}n\sum_{\begin{array}{c} i,j=1\\ i+j=n \end{array}}^{L}P_{ij}$$

Sum of entropy:

$$SH=-\sum_{n=2}^{2L}\sum_{\begin{array}{c} i,j=1\\ i+j=n \end{array}}^{L}P_{ij}\;\log\left(\sum_{\begin{array}{c} i,j=1\\ i+j=n \end{array}}^{L}P_{ij}\right)$$

Difference of entropy:

$$DH=-\sum_{n=0}^{L-1}\sum_{\begin{array}{c} i,j=1\\ |i-j|=n \end{array}}^{L}P_{ij}\;\log\;\left(\sum_{\begin{array}{c} i,j=1\\ |i-j|=n \end{array}}^{L}P_{ij}\right)$$

Sum of variance:

$$SV=\sum_{n=2}^{2L}{\left(n-SH\right)}^{2}\sum_{\begin{array}{c} i,j=1\\ i+j=n \end{array}}^{L}P_{ij}$$

Difference of variance:

$$DV=\mathrm{var}\left\{\sum_{\begin{array}{c} i,j=1\\ |i-j|=n \end{array}}^{L}P_{ij}\right\}$$

Maximum probability:

$$M=\underset{i,j}{\max}P_{ij}$$

Maximal Correlation Coefficient:

$$MCC=2^{\mathrm{nd}}\mathrm{eig}\left\{Q\right\}$$

where

$$Q=\frac{\sum_{k}P_{ik}P_{jk}}{\sum_{j}P_{ij}\sum_{i}P_{ik}}$$

and 2^*nd*^ eig refers to the second eigenvalue of *Q*.

Information measures of correlation

$$IC1=\frac{H-{H1}_{XY}}{\max\left\{H_{X},H_{X}\right\}}$$

$$IC2={\left(1-\exp\left(-2({H2}_{XY}-H)\right)\right)}^{2}$$

where

$$H_{X}=-\sum_{i}(\sum_{j}P_{ij})\log\left(\sum_{j}P_{ij}\right)$$

$$H_{Y}=-\sum_{j}(\sum_{i}P_{ij})\log\left(\sum_{i}P_{ij}\right)$$

$${H1}_{XY}=-\sum_{i,j}P_{ij}\log\left(\sum_{i}P_{ij}\sum_{j}P_{ij}\right)$$

$${H2}_{XY}=-\sum_{i,j}(\sum_{i}P_{ij}\sum_{j}P_{ij})\log\left(\sum_{i}P_{ij}\sum_{j}P_{ij}\right)$$
